# Under-reported COVID-19 cases in South Asian countries

**DOI:** 10.12688/f1000research.36705.2

**Published:** 2021-03-17

**Authors:** Soban Qadir Khan, Imran Alam Moheet, Faraz Ahmed Farooqi, Muhanad Alhareky, Faisal Alonaizan

**Affiliations:** 1 Department of Dental Education College of Dentistry, Imam Abdulrahman Bin Faisal University (IAU), Dammam, Saudi Arabia; 2 Department of Science Dental Material, Azra Naheed Dental College, Superior University, Lahore, Pakistan; 3 Department of Science Dental Material, Baqai Dental College, Baqai Medical University, Karachi, Pakistan; 4 Department of Academic Affairs, College of Dentistry, Imam Abdulrahman Bin Faisal University, Dammam, Saudi Arabia; 5 Department of Dental Education, College of Dentistry, Imam Abdulrahman Bin Faisal University, Dammam, Saudi Arabia; 6 Department of Preventive Dental Sciences, College of Dentistry, Imam Abdulrahman Bin Faisal University, Dammam, Saudi Arabia; 7 Department of Restorative Dental Sciences, College of Dentistry, Imam Abdulrahman Bin Faisal University, Dammam, Saudi Arabia

**Keywords:** COVID-19, coronavirus disease, SARS-CoV-2, South Asia, pandemic

## Abstract

**Background: **The ****purpose of the study was to compare  trends in the progression of COVID-19 among South Asian countries with more developed Western countries.

**Methods: **COVID-19 data from South Asian countries were used for this observational study. Data were taken up to April 21, 2020 from the outbreak of the COVID-19. Four of the seven countries met the inclusion criteria and were included in the analysis.

**Results: **An exponential increase in the average number of weekly cases was reported after the fifth week following the first case. The correlation between reported cases and tests was found to be strong and significant (r=0.90, p=0.037). However, on average, 315.25 tests per million population were performed, which was at least 12 times lower than the number of tests performed in countries with a large number of COVID-19 cases.

**Conclusions: **At present, the number of confirmed cases from South Asia was found to be significantly lower than in Western countries. Hence, an increase in the strength of performing diagnostic tests is highly recommended. Strict measures are required to make the people of these countries follow the instructions of social distancing and comply with preventive measures.

## Introduction

Any outbreak of an infectious disease or a natural disaster on a large scale, which spreads over a large geographical area leading to morbidity and mortality, is known as a pandemic. Evidence suggests that the likelihood of pandemics has increased over the past century because of an increase in global travel, urbanization, and greater exploitation of the natural environment ^1^. Consequences of pandemics are multidimensional, have an impact on global health, socioeconomic conditions, and political implications ^2^. The recent outbreak of coronavirus disease (COVID-19) was reported in the Huanan Seafood Market in Wuhan, China. China is therefore considered as the epicenter of the disease ^3^. However, in the current scenario, some European countries and the United States of America (USA) have become new epicenters of the disease. Individually, these countries have more than twice the number of reported cases when compared to China. Meanwhile, their death toll is at least five times greater than in China ^4^. During the second week of March, the COVID-19 outbreak was declared a pandemic by the World Health Organization (WHO) ^5^.

The severe acute respiratory syndrome coronavirus 2 (SARS-CoV-2) that causes COVID-19, belongs to the same family of corona viruses that includes the Middle East respiratory syndrome virus (MERS-CoV) ^6^. Signs and symptoms of COVID-19 include respiratory symptoms, fever, cough, and shortness of breath. These breathing difficulties can worsen over time, and the disease can lead to complications such as: pneumonia, severe acute respiratory syndrome, kidney failure, and even death. People with low immunity and underlying systemic diseases are more prone to SARS-CoV-2 infection ^7^. This is why the highest death rates are reported in the elderly population among those who were infected by COVID-19 ^8^.

Various precautionary methods have been adapted by countries to control and stop the spread of COVID-19. A few such measures include hygiene maintenance, public awareness, partial lockdown, complete lockdown, and even the imposition of curfews ^9^. Currently, social distancing is the only recognized way to prevent the spread of the virus. Hence, countries are making decisions based on their circumstances and the experiences of other countries ^10^. Based on data extracted from the WHO database, the progression of the disease and deaths in various regions and countries differ ^11^. Statistics show a high number of reported COVID-19 cases and deaths in some European countries and in the USA as well. The first confirmed case of COVID-19 in some South Asian countries like India and Sri Lanka was reported on 15 ^th^ of February 2020 while in Pakistan and Bangladesh, the first confirmed case was reported on 26 ^th^ February and 8 ^th^ March respectively. The trend in average weekly increases in reported cases remained untested. Therefore, the objective of the current study was to find the trend in the progression of COVID-19 among South Asian countries compared to more developed Western countries.

## Methods

This observational study was conducted between 20 ^th^ April and 22 ^nd^ April 2020. The study included COVID-19 data available from worldometer® ^12^. Data was extracted from the date of the first COVID-19 case reported up until 21 ^st^ April 2020. Data was extracted for all South Asian countries that were affected by the current outbreak. Only countries with at least 100 reported cases by 21 ^st^ April 2020 were included in the study and reason to do so was to prevent the analysis process from outliers’ effects as well as to keep pictorial presentation visualized. Hence, a total of four out of the seven countries were included in the study: India, Pakistan, Sri Lanka, and Bangladesh.

The variables taken from the data source were: (1) total reported cases, (2) total deaths, (3) total recovered, (4) number of cases with outcome, (5) number of serious/critical cases, (6) total cases per one million population, (7) total tests performed, and (8) total tests performed per one million population. Furthermore, a few more variables were calculated using the extracted data and variables. The total outcome was calculated by adding the total number of deaths and the total number of recoveries. Percentage of deaths was calculated by using the equation [total deaths / (total deaths reported + total recovered) x 100%], the percentage of recovered cases was calculated as [total recovered/ (total deaths reported + total recovered) x 100%], the percentage of critical cases as “number of critical cases/active cases × 100%”, and the ratio of the number of cases tested by dividing the total number of cases by the total number of tests performed. The weekly number of cases reported after the first case reported until 21 ^st^ April 2020 was also extracted from the data source. For the descriptive comparison of the present study’s findings of countries with a high number of COVID-19 cases, some of the statistics are summarized in Table 1 ^10^.

**Table 1.** Data for COVID-19 extracted from worldometer® ^10^.

Countries	No. of reported cases	No. of deaths reported	No. of recovered cases	No. of tests performed per million	Population (per km ^2^)
USA	792,938	42,518	72,389	12,167	36
Spain	204,178	21,282	82,514	19,896	94
Italy	181,228	24,114	48,877	23,122	206
France	155,383	20,265	37,409	7,103	119
UK	124,743	16,509	N/A	7,386	75
China	82,758	4,632	77,123		153
Turkey	90,980	2,140	13,430	7,991	110
Iran	83,505	5,209	59,273	4,203	52

The statistical package for social sciences (SPSS v. 23) was used for the analysis. Descriptive statistics included the calculation of averages and standard deviations as well as line graphs to present the number of weekly reported cases in each country. In inferential statistics, a simple linear regression was used between total cases (dependent variable) and total tests performed (independent variable). The Wilcoxon signed-ranks test was used to analyze the weekly increase in COVID-19 cases.

## Results

The total number of reported COVID-19 cases in South Asian countries by 21 ^st^ April 2020 were 31,565 of which 5,526 (17.5%) recovered. The number of reported deaths was 901. Among South Asian countries, India had the highest number of positive COVID-19 cases (18,658; 59.1%), followed by Pakistan (9,216; 29.2%), Bangladesh (3,382; 10.7%), and Sri Lanka (309; 1%) ( Table 2). The percentage of reported deaths was highest in Bangladesh (55.84%), followed by India (15.32%), Sri Lanka (6.54%), and Pakistan (8.5%).

**Table 2.** Country-wise extracted and computed variables after inclusion criteria for South Asian countries.

Countries	Cases	Deaths	Recovered	OUTCOME	Death (%)	Recovered (%)	No. of tests performed per million pop.
India	18,658	592	3,273	3,865	15.32	84.68	291
Pakistan	9,216	192	2,066	2,258	8.50	91.50	506
Sri Lanka	309	7	100	107	6.54	93.46	302
Bangladesh	3,382	110	87	197	55.84	44.16	162

**Figure 1.**
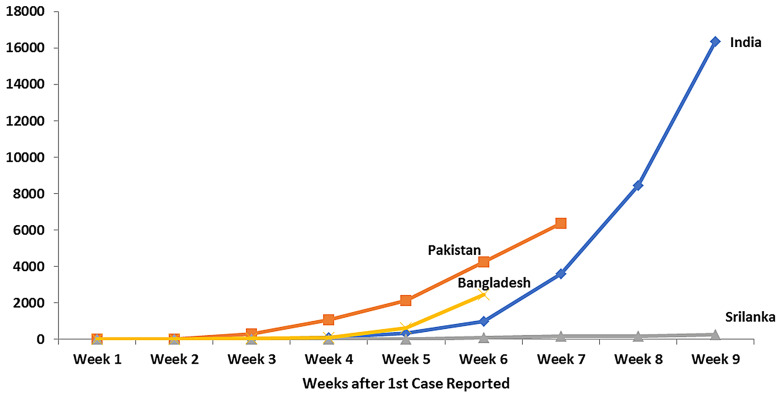
Weekly comparison of reported COVID-19 cases.

**Figure 2.**
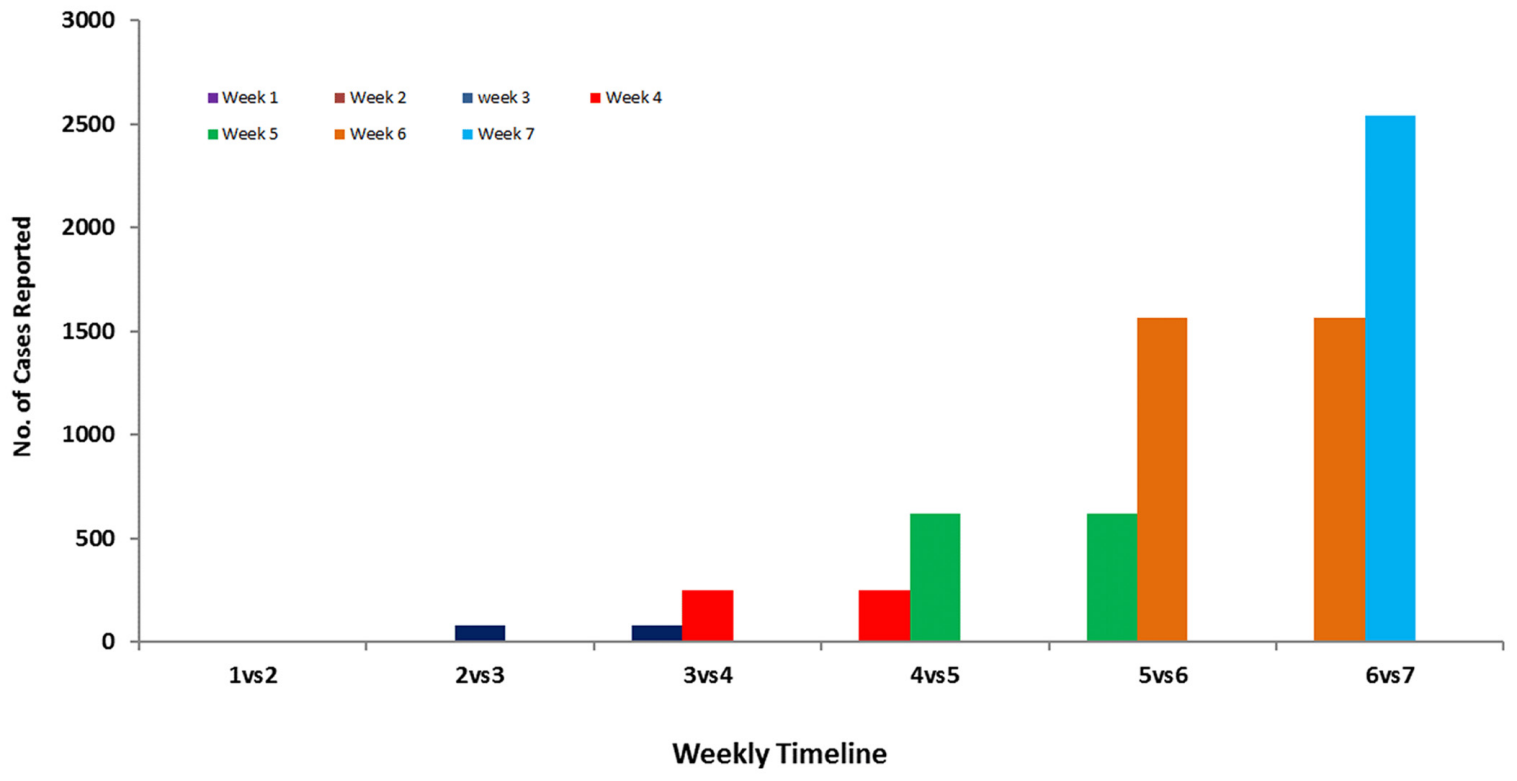
Weekly comparison of reported cases for South Asian countries.

Figure 1 shows the exponential growth in the number of reported cases among the South Asian countries after the fourth to fifth week since the start of the disease. A sharp increase was observed in reported cases in India from the sixth week onwards. In Pakistan, the number of reported cases was also found to increase at the start of the fourth week. However, the spread of the virus was not as rapid in Bangladesh and Sri Lanka, as was found in the Indian and Pakistani populations. A comparison of the average number of reported cases in each week with the previous week revealed that the average increase in cases was not statistically significant except between fifth and sixth week (z-value -2.02, p-value 0.043). Figure 2 presented descriptive comparison of the average number of confirmed cases between two consecutive weeks.

On average, 315.25 tests per million population were performed in the countries included in the current study. A correlation between the total reported cases and the total number of tests performed was found. The data revealed a very strong, direct, and statistically significant correlation (r=0.90, p=0.037). The Bangladeshi population had the highest percentage of tested positive cases in relation to the total number of tests performed (12.71%), followed by Pakistan (8.24%), Sri Lanka (4.78%), and India (4.65%).

The regression model between the number of tests performed (independent variable) and total number of cases reported (dependent variable) demonstrated a very strong R-square value 0.925 with significance of the model (p =0.025). The predicted values of constant and slope for the model were 1442.4 and 0.045, respectively. Hence, the regression equation can be written as

No. of cases reported = 1442.4 + 0.045 (No. of tests performed)

## Discussion

This study particularly highlighted the importance of maintaining data of COVID-19 patients and it indicated underestimation of COVID-19 positive cases in South Asian countries. Another important aspect of the findings was it regressed a model which found number of tests performed had significant and strong relation with number of cases. By comparing Table 1 and Table 2, difference in number of tests performed (per million) among countries with high number of COVID-19 cases ( Table 1) and in South Asian countries ( Table 2) can be seen. 

As of 21 ^st^ April 2020, there had been over 2.5 million reported cases of COVID-19 in 210 countries across six continents. At the beginning of this pandemic, China (82,758) was affected the most by the disease; however, later the USA (792,938), Spain (204,178), Italy (181,228), France (155,383), Turkey (90,980), and Iran (83,505) had the most COVID-19 cases worldwide. The number of reported cases has since started to increase in South Asian countries (India, Pakistan, Sri Lanka, and Bangladesh). The initial cases reported in South Asian countries were thought to be caused by travelers returning from other COVID-19 affected countries. Although, by the date (21 ^st^ April, 2020), the total number of reported cases in South Asian countries is not as high as it was in the USA, Italy, Spain, France, and Iran ( Table 1). However, the weekly growth in the number of reported cases (up to the eighth week) in South Asian countries is quite similar to the increase in the number of reported cases in the USA, France, and the United Kingdom (UK).

Based on the preparedness index formulated by Greenhill and Oppenheim, which defines the ability of a country to curtail any pandemic ^13^, the spread risk of this pandemic is higher in South Asian developing countries than in developed countries ^14^. A few of the factors that contribute to a higher spread risk of the pandemic include population density, susceptibility to infection, patterns of movement driven by travel, trade, and migration, the speed and effectiveness of public health surveillance and response measures, and the socioeconomic status of the country ^14^. Three out of four countries included in the current study fall under the top ten most populated countries in the world ^15– 17^. Furthermore, the per km ^2^ population in Bangladesh, India, and Pakistan is more than any country listed in Table 1. In addition, a large number of people in these countries live in slums ^15– 17^, which makes it difficult to maintain social distancing and to adopt preventive measures. Furthermore, poor education and extreme poverty are other factors that make it more difficult to follow social distancing instructions, or early disease identification of symptoms of COVID-19. A report from Pakistan showed an increasing number of cases where people died due to COVID-19 before reaching a hospital ^18^.

To date, there has been a total of 2,505,858 reported cases of COVID-19 worldwide, with a lower number of reported cases (31,608) and reported deaths (901) in South Asian countries compared to other regions of the world. There have been many hypotheses related to this lower reported COVID-19 cases and deaths. Some of the theories included stronger immunity, warmer weather, childhood BCG vaccinations, and exposure to anti-malaria medications. From the data collected from the worldometer® website, the authors believe that the lower number of reported cases in the South Asian countries could be due to the lower number of diagnostic tests performed for COVID-19 virus compared to countries that have reported a higher number of COVID-19 cases. India (18,658) and Pakistan (9,216) have the most COVID-19 cases in South Asian countries, with 291 and 506 tests performed per million population, respectively. The number of tests (per million) performed in India and Pakistan is significantly lower than in the USA (12,167), Spain (19,896), Italy (23,122), France (7,103), and the UK (7,386). Even countries in Asia with the highest number of COVID-19 cases, Turkey (7,991) and Iran (4,203), have a higher number of tests performed per million population. In South Asia, on average, 315.25 tests per million population were performed; this is at least 12 times lower than the number of tests performed in the epicenters of COVID-19.

Even though the world is better prepared to face any health crisis than before, still the world is not fully prepared to handle a pandemic. Preparedness for any crisis does affect from financial status of any country hence financially strong countries would have better chances to have more health care facilities, resources, infrastructures to cope with any crisis. In present time, world is facing a pandemic and so far, its deep impact can be seen in various continents however it is not significantly hit south Asian countries yet.

Accountability for preparedness in these countries is diffuse, and many countries that are at the greatest risk have the most limited capacity to manage and mitigate pandemic risk. In addition, these countries need to perform the virus diagnostic tests in greater numbers to get an accurate picture of the pandemic. Based on the data, one could suggest that the low number of reported cases but with high percentage increases for South Asian countries could be a ticking time bomb waiting to explode, and this region could be the next highlighted region of this current pandemic.

## Conclusions

Although the current number of reported cases and reported deaths from South Asia suggested that the spread of COVID-19 is not as high as it was in many other countries. However, a comparison of statistics and population characteristics does not portray a good picture for the future. Therefore, the following conclusions can be drawn:

- **1.** One side of the picture is a lower number of reported cases and deaths, but the other side of the picture suggests a large number of cases that are prevailing in the society that are unidentified and undiagnosed. Hence, identifying the spread of the disease by increasing the number of diagnostic tests is highly recommended.
- **2.** Governments in these countries are required to take strict measures such as partial or complete lockdowns in order to maintain proper social distancing, since a high population density coupled with low education levels and low disease awareness could lead to a new disease epicenter.

## Data availability

### Underlying data

Harvard Dataverse: Low Reported COVID-19 Cases in South Asian Countries: A Luck of Nature or A Ticking Time Bomb, https://doi.org/10.7910/DVN/QLTVRW ^19^.

Data are available under the terms of the Creative Commons Zero “No rights reserved” data waiver (CC0 1.0 Public domain dedication).

## Author information

Imran Alam Moheet was affiliated with Azra Naheed Dental College, Superior University, Pakistan at the time this paper was written but is currently at Baqai Dental College, Baqai Medical University, Pakistan. Faraz Ahmed Farooqi was affiliated with Department of Academic Affairs, Imam Abdulrahman Bin Faisal University, Saudi Arabia at the time this paper was written but is currently affiliated with Department of Dental Education, Imam Abdulrahman Bin Faisal University, Saudi Arabia.
