# Supplementary material for: Under-reported COVID-19 cases in South Asian countries
Source: F1000Res. 2021 Mar 17;10:88. Originally published 2021 Feb 10. [Version 2] doi: 10.12688/f1000research.36705.2 (PMC7905493; doi:10.12688/f1000research.36705.2)
Supplement: Peer review history

# Peer review: Reviewer response for version 2

**DOI:** 10.5256/f1000research.54941.r81572

**Published:** 2021-03-22

**Authors:** Khalid Aziz Ansari

**Affiliations:** 1 Department of Physiology, University of the West of England, Bristol, UK

Author addresses all my comments comprehensively and the manuscript is now looks fine overall and can be indexed.

Is the work clearly and accurately presented and does it cite the current literature?

Partly

If applicable, is the statistical analysis and its interpretation appropriate?

I cannot comment. A qualified statistician is required.

Are all the source data underlying the results available to ensure full reproducibility?

Partly

Is the study design appropriate and is the work technically sound?

Yes

Are the conclusions drawn adequately supported by the results?

Yes

Are sufficient details of methods and analysis provided to allow replication by others?

Yes

Reviewer Expertise:

Acute and Chronic lung diseases, epidemiology

I confirm that I have read this submission and believe that I have an appropriate level of expertise to confirm that it is of an acceptable scientific standard.

# Peer review: Reviewer response for version 2

**DOI:** 10.5256/f1000research.54941.r81573

**Published:** 2021-03-18

**Authors:** Junaid Ahmed, Nanditha Sujir

**Affiliations:** 1 Department of Oral Medicine and Radiology, Manipal College of Dental Sciences, Manipal Academy of Higher Education, Mangalore, India

The revised manuscript has been noted with no additional comments.

Is the work clearly and accurately presented and does it cite the current literature?

Yes

If applicable, is the statistical analysis and its interpretation appropriate?

I cannot comment. A qualified statistician is required.

Are all the source data underlying the results available to ensure full reproducibility?

Yes

Is the study design appropriate and is the work technically sound?

Yes

Are the conclusions drawn adequately supported by the results?

Partly

Are sufficient details of methods and analysis provided to allow replication by others?

Yes

Reviewer Expertise:

NA

We confirm that we have read this submission and believe that we have an appropriate level of expertise to confirm that it is of an acceptable scientific standard.

# Peer review: Reviewer response for version 1

**DOI:** 10.5256/f1000research.39768.r79261

**Published:** 2021-02-24

**Authors:** Junaid Ahmed, Nanditha Sujir

**Affiliations:** 1 Department of Oral Medicine and Radiology, Manipal College of Dental Sciences, Manipal Academy of Higher Education, Mangalore, India

Thank you for the opportunity to review this article. This article is related to the most pressing health crises faced by mankind i.e COVID-19. The article is well written and adheres to the formatting of the journal.

 Few points need clarification,

1. In the methodology, the authors state that only data from South Asian countries have been included. However, the data from other developed countries have also been included for comparison.
2. Discussion:
  a) Kindly avoid restating the results in the discussion section.
  b) The authors should discuss the variations in the COVID-19 testing policies that the countries adopted initially , as it is variable from country to country and can alter the number of tests. Also, in the discussion the authors should elaborate on the timings and implementation of the country wide lockdown in the countries, that could have affected the rate of spread of COVID-19.

Is the work clearly and accurately presented and does it cite the current literature?

Yes

If applicable, is the statistical analysis and its interpretation appropriate?

I cannot comment. A qualified statistician is required.

Are all the source data underlying the results available to ensure full reproducibility?

Yes

Is the study design appropriate and is the work technically sound?

Yes

Are the conclusions drawn adequately supported by the results?

Partly

Are sufficient details of methods and analysis provided to allow replication by others?

Yes

Reviewer Expertise:

NA

We confirm that we have read this submission and believe that we have an appropriate level of expertise to confirm that it is of an acceptable scientific standard, however we have significant reservations, as outlined above.

# Peer review: Reviewer response for version 1

**DOI:** 10.5256/f1000research.39768.r79263

**Published:** 2021-02-24

**Authors:** Sumanth Kumbargere Nagraj

**Affiliations:** 1 Department of Oral Medicine and Oral Radiology, Melaka-Manipal Medical College, Melaka, Malaysia

This is an observational study based on the data taken up to 21st April 2020. During this period South Asian countries mentioned in this study were already under lockdown (Bangladesh: from 22 March to 30 May; India: 24 March to 31 May and Sri Lanka: 26 March to 11 May). I do not understand why the authors conclude by suggesting lockdowns as their only suggestion. In contrast to their suggestion, today, these are the countries which are doing better compared to the countries which have or are still under lockdowns.

 The authors have not considered the issues related to the population of India or Pakistan, the handful testing centres these countries had in the month of April with limited number of test kits and the availability of PPE kits in these four countries. Comparing a population of 1.3 billion (India) or 216 million (Pakistan) to countries with fraction of its population and suggesting lockdown looks futile. The economic impact of lockdown is another aspect which needs to be weighed before opting for a lockdown in these countries. 

 However, the article highlights the importance of social distancing and preventive measures that needs to be implemented in these countries which are the most economic preventive measures.

Is the work clearly and accurately presented and does it cite the current literature?

Yes

If applicable, is the statistical analysis and its interpretation appropriate?

Partly

Are all the source data underlying the results available to ensure full reproducibility?

Yes

Is the study design appropriate and is the work technically sound?

Partly

Are the conclusions drawn adequately supported by the results?

Yes

Are sufficient details of methods and analysis provided to allow replication by others?

Yes

Reviewer Expertise:

Evidence-based healthcare, qualitative evidence synthesis, Randomised controlled trials in Oral healthcare, Research priority setting

I confirm that I have read this submission and believe that I have an appropriate level of expertise to confirm that it is of an acceptable scientific standard.

# Peer review: Reviewer response for version 1

**DOI:** 10.5256/f1000research.39768.r79264

**Published:** 2021-02-15

**Authors:** Khalid Aziz Ansari

**Affiliations:** 1 Department of Physiology, University of the West of England, Bristol, UK

This is interesting study and following areas need a little more revision.

**Introduction section:**

 There are some missing references. (" *The recent outbreak...epicentres of the disease, Currently, social distancing is the only recognized way to prevent the spread of the virus. Hence, countries are making decisions based on their circumstances and the experiences of other countries...of other countries"*).

**Method:**

 To add more clarity, it needs a little more justification on why countries with more than 100 cases were selected.

**Discussion:**

 This section need major revision in relation to organisation. It does not clearly reflects the results of the study and the impact of present study findings in the these countries. For example, which of your study findings suggest that this disease is zoonotic?

Is the work clearly and accurately presented and does it cite the current literature?

Partly

If applicable, is the statistical analysis and its interpretation appropriate?

I cannot comment. A qualified statistician is required.

Are all the source data underlying the results available to ensure full reproducibility?

Partly

Is the study design appropriate and is the work technically sound?

Yes

Are the conclusions drawn adequately supported by the results?

Yes

Are sufficient details of methods and analysis provided to allow replication by others?

Yes

Reviewer Expertise:

Acute and Chronic lung diseases, epidemiology

I confirm that I have read this submission and believe that I have an appropriate level of expertise to confirm that it is of an acceptable scientific standard, however I have significant reservations, as outlined above.

# Response

**Published:** 2021-02-24

**Authors:** soban khan

**Affiliations:** Imam abdulrahman bin Faisal University, Saudi Arabia

Dear Khalid Aziz,

 Thank you for your time you gave to review this article. I appreciate your comments which will help to improve presentation of our work. We have modified the manuscript to address your comments. 

 Many Thanks.
